# Iodine Status Has No Impact on Thyroid Function in Early Healthy Pregnancy

**DOI:** 10.1155/2012/168764

**Published:** 2012-11-28

**Authors:** F. Brucker-Davis, P. Ferrari, J. Gal, F. Berthier, P. Fenichel, S. Hieronimus

**Affiliations:** ^1^Department of Endocrinology, Diabetology and Reproductive Medicine, l'Archet Hospital, CHU de Nice, 151 route de Saint-Antoine, 06200 Nice, France; ^2^Department of Biochemistry, CHU de Nice, 151 route de Saint-Antoine, 06200 Nice, France; ^3^Department of Biostatistics, CHU de Nice, 151 route de Saint-Antoine, 06200 Nice, France

## Abstract

*Aim*. To assess the impact of iodine status in early pregnancy on thyroid function. *Methods*. Women >18 years old seen at their first prenatal consult before 12 weeks of amenorrhea and without personal thyroid history were proposed thyroid screening and were eligible if they had strictly normal thyroid tests (fT4 > 10th percentile, TSH < 2.5 mUI/L, negative anti-TPO antibodies). Evaluation included thyroid ultrasound, extensive thyroid tests, and ioduria (UIE). *Results*. 110 women (27.5 y, 8 weeks of amenorrhea, smoking status: 28% current smokers) were enrolled. Results are expressed as medians. UIE was 116 **μ**g/L. 66.3% of women had iodine deficiency (ID) defined as UIE < 150. FT4 was 14.35 pmol/L; TSH 1.18 mUI/L; fT3 5 pmol/L; thyroglobulin 17.4 ng/mL; rT3 0.27 ng/mL; thyroid volume: 9.4 ml. UIE did not correlate with any thyroid tests, but correlated negatively with thyroid volume. UIE and all thyroid tests, except fT3, correlated strongly with **β**hCG. Smoking correlated with higher thyroid volume and thyroglobulin and with lower rT3. *Conclusions*. In pregnant women selected for normal thyroid function, mild ID is present in 66% during the 1st trimester. The absence of correlation between UIE and thyroid tests at that stage contrasts with the impact of **β**hCG and, to a lesser degree, maternal smoking.

## 1. Introduction

Mild iodine deficiency (ID) is still common in western Europe, despite government programs aiming at its eradication, usually based on salt iodination. Diagnosis of ID still rests on spot ioduria (UIE) at the population level, though this tool is imperfect [1]. We have previously shown in a cross-sectional study that the prevalence of ID in pregnant women of our area was present during the 3d trimester in more than three-quarter of cases [2].

While severe ID is associated with dramatic impairment of neurocognitive development of the offspring, the deleterious developmental impact of mild to moderate ID is less documented [3, 4]. It could be mediated by fetal ID per se or via maternal hypothyroxinemia or hypothyroidism that may develop in predisposed women during pregnancy [5–7]. The changes of thyroid economy throughout pregnancy include a drop in fT4 after an early phase of thyroid stimulation [8]. However, the definition of maternal hypothyroxinemia is debated [5, 9]. The benefit of iodine supplementation is documented for women selected for thyroid hyperstimulation in early pregnancy and their offspring, based on fetal thyroglobulin (Tg) [10], or in case of severe to moderate ID based on neurodevelopmental evaluation [3, 11, 12]. However, there is no definitive evidence of a benefit both in terms of maternal thyroid function and of neurocognitive development of her offspring in healthy pregnant women with normal thyroid function at the beginning of pregnancy [13]. 

We report here results assessing the impact of iodine status on thyroid function during the first trimester of pregnancy in healthy women.

## 2. Patients and Methods

### 2.1. Patients

In order to select pregnant women with normal thyroid tests, we proposed a thyroid screen to all women who age > 18 years, without personal history of thyroid disease consulting at the obstetric clinic of our hospital before 12 weeks of amenorrhea (WA) with a singleton pregnancy between July 2007 and July 2008. The screening evaluation included free T4, TSH, and antithyroperoxidase (TPO) antibodies. For this study, we specifically recruited women who were not taking iodine supplementation. A spot urine sample was also collected to allow UIE measurement in case of participation to the study. We included 110 TPO-negative women with strict criteria of normal thyroid tests who agreed to participate and signed a consent form. Ranges for normal thyroid tests in our laboratory for the first trimester are (2.5–97.5 percentile of TPO negative women) fT4: 11.47–19.3 pmol/L; TSH: 0.053–3.23 mUL/L. We selected women with fT4 > 10th percentile (12 pmol/L) and TSH < 2.5 mUL/mL, as that threshold is currently recommended for pregnant women [14]. 

Maternal smoking was assessed qualitatively by self-reported statement: never smoked, current smokers, and former smokers (regroup women who quit smoking before pregnancy or when diagnosed as pregnant). 

Thyroid ultrasound was performed by two of us (F. Barker Davis and S. Hieronimus) who concerted and agreed before the study about thyroid volume measurement criteria using an En Visor scanner (Philips Medical System) equipped with a commercially available from 5 to 12 MHz linear transducer (50 mm length). Volume was calculated in mL for each lobe according to the formula 0.52 × height × width × thickness in centimeters. Total thyroid volume was the sum of each lobe's volume, the isthmus was not taken into account in volume calculation. 

### 2.2. Assays

Spot UIE was measured by mass spectrometry ICP/MS (Pasteur-Cerba Laboratory, Cergy Pontoise, France, detection threshold 15 *μ*g/L; intra- and interseries CV < 10%). Free T4 (fT4), free T3 (fT3), total T4 (TT4), TSH, *β*hCG, and anti-TPO and anti-Tg antibodies were measured by chemiluminescence (ADVIA Centaur, Siemens Healthcare Diagnostics, France). Tg was measured by immunoradiometric assay (Thyroglobulin IRMA, Cis bio International, Gif sur Yvette, France). Thyroxin binding globulin (TBG) and reverse T3 (rT3) were measured by radio-immunoassay (RIA): RIA-gnost TBG (Cis bio International, Gif sur Yvette, France) and RIA rT3 (Pasteur Cerba Laboratory, Cergy Pontoise, France). Reference ranges outside pregnancy, intra- and intercoefficients of variation were as the following: fT4 9–23 pmol/L (intra-assay coefficient of variation (CV) 2.31%; interassay CV 1.95%); fT3 3–7 pmol/L (intra-assay CV 2.35); TT4 during pregnancy 82.6–138 nmol/L (intra-assay CV 1.77%; interassay CV 2.9%); TSH 0.1–4 mUI/L (intra-assay CV 2.67%; interassay CV 3.97%); *β*hCG < 10 UI/L (intra-assay CV 2.8%; interassay CV 4.3%); TPO antibodies < 100 UI/mL (intra-assay CV 4.1%, interassay CV 8.0%); Tg antibodies < 60 UI/mL (intra-assay CV 5.5%; interassay CV 1.8%); Tg 5–50 ng/mL (intra-assay CV 2.4%; interassay CV 4.5%); rT3 0.14–0.54 nmol/L (intra-assay CV 8.54% interassay CV 6.21%); reference range for TBG for the first trimester 20.5 ± 4.8 *μ*g/mL (interassay CV 4.4%).

### 2.3. Statistics

Quantitative variables are expressed as means and standard deviation, medians, and range. Qualitative variables are expressed as counts and percentages. Student's *t* test or Mann-Whitney's *U* test were used to compare continuous variables. Chi-2 test or Fisher's exact test were used for categorical variables. The correlations between variables were determined using the Pearson's test or Spearman's rank test. The nonparametric Kruskal-Wallis test was used to examine associations between maternal smoking and thyroid function tests.

Statistical analyses were performed using the software SAS version 9.1. Values were considered significant when *P* < 0.05. All tests were two-sided.

## 3. Results

### 3.1. Clinical Characteristics of the 110 Included Women

Their median age was 27.5 y (18–40), their body mass index (BMI) was 22.4 (16–45.3), with a weight at inclusion of 61.5 kg (43–128 kg), and they were nulliparous in 44.5%. 53% had never smoked, 18% were former smokers, and 28% were current smokers. 15% had a family history of thyroid disease; 82% were born in France (including 56% in our area). Thyroid ultrasound allowed the detection of 16 nodules (14.6%), including six women with nodules >1 cm. Global thyroid volume was 9.4 mL (4.5–17.9 mL); thus, no patient had thyroid hyperplasia or goiter. 

### 3.2. UIE and Thyroid Tests (Table 1)

**Figure 1:**
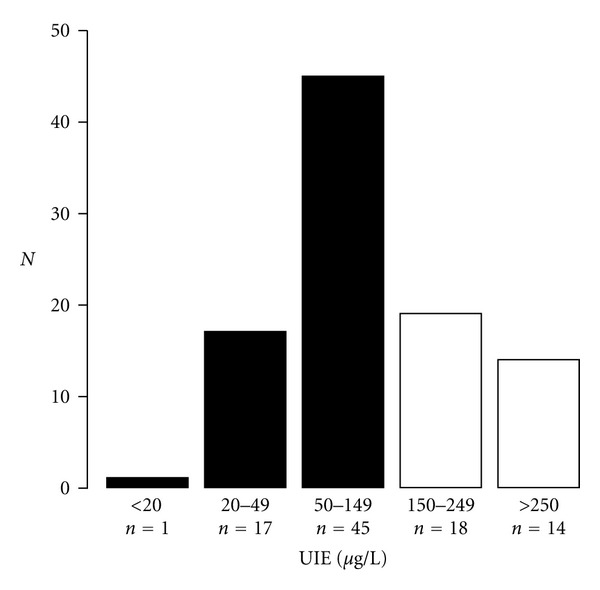
Distribution of ioduria (UIE) in healthy euthyroid pregnant women. Solid black histograms represent women (*n* = 63 of 95) with UIE reflecting ID according to the latest WHO guidelines (UIE < 150 *μ*g/L).

Median ioduria was 116 *μ*g/L. The repartition of the population according to WHO criteria during pregnancy (WHO) for iodine status (15) is shown in Figure 1: 1% (*n* = 1) with severe ID <20 *μ*g/L; 18% (*n* = 17) with moderate iodine deficiency, UIE between 20 and 50 *μ*g/L; 47% (*n* = 45) with mild ID, UIE between 50 and 149 *μ*g/L; 19% (*n* = 18) with adequate iodine intake, UIE between 150–250 *μ*g/L; 15% (*n* = 14) with more than adequate iodine intake. With a threshold of UIE at 150 *μ*g/L, 66% of pregnant women seen early were considered iodine deficient. There was no seasonal variation in UIE (data not shown). Women with ID based on UIE < 150 *μ*g/L tended to have higher thyroid volumes (*n* = 61, median 10 mL) compared to women with UIE between 150 and 250 *μ*g/L (*n* = 19, median 8.9 mL), while women with UIE > 250 mcg/L had intermediate volume (*n* = 14, median 9.4 mL).

Table 1 summarizes all thyroid tests. Among those women with normal thyroid tests, seven had a Tg > 50 ng/L (upper limit of normal for nonpregnant women). Those seven women had higher TSH (1.73 versus 1.15 mUI/L, *P* = 0.04), less homogenous thyroid on ultrasound (four out of seven, versus nine out of 103, *P* < 0.0001) and were more often smokers (five out of seven, versus 24 out of 103, *P* = 0.0006). Last, women who later had a miscarriage (*n* = 7) had lower *β*hCG (9930 versus 66704 UI/L, *P* = 0.005) and lower TT4 (94.9 versus 111.6 pmol/L, *P* = 0.04).

### 3.3. Correlations (Table 2)

UIE did not correlate with any of the thyroid tests. However, it correlated negatively with thyroid volume and maternal age, but not with parity nor gravidity. It correlated positively with *β*HCG. 

Thyroid tests correlated strongly with term (data not shown) and even more with *β*hCG: *β*hCG correlated negatively with TSH and positively with TT4, rT3, and TBG, and marginally with fT4. Maternal weight tended to correlate negatively with fT4 and positively with TSH. There was no correlation of thyroid volume with thyroid tests, including Tg (data not shown).

Maternal smoking was associated with higher thyroid volume (smokers 10.6 mL versus 9.3 mL in nonsmokers, *P* = 0.04) and higher Tg (smokers 30.6 versus nonsmokers 14.8 ng/mL, *P* = 0.0005), and with lower rT3 (smokers 0.2 versus nonsmokers 0.3 nmol/L, *P* = 0.01).

## 4. Discussion

We have selected women with strictly normal thyroid function, in choosing stringent criteria (above 10th percentile of our trimester specific reference range for fT4 and <2.5 mUI/L for TSH). There was no goiter nor thyroid hypertrophy as assessed by ultrasound. Thyroid volume in our population was smaller than the volume observed by Glinoer et al. (14.3 mL) in his population with “stimulated” thyroid [10]. The rate of thyroid incidentaloma was similar to the rate observed in the general population [15]. 

The median UIE in our population with no iodine supplementation was 116 *μ*g/L, which is below the currently recommended threshold in pregnancy (150 *μ*g/L), in the range of mild iodine deficiency [16]. While spot ioduria is useless to establish the diagnosis of ID at an individual level, given its variation from day to day [1], so far it remains the gold standard at the population level [17]. There is a wide range of published UIE values in pregnancy, depending in part on national public health policies to tackle iodine deficiency. Another important variation factor is gestational age, with a steady decline from the first trimester to the end of pregnancy [18]. Indeed, the apparent better iodine status in early pregnancy may be instead due to the increase of glomerular filtration during first trimester causing an increased UIE [19] and, thus, an increased loss of iodine. Two previous studies in France, including one from our Department, have shown lower UIE values at the end of pregnancy: 54 *μ*g/L [20] and 64 *μ*g/L [2]. When focusing on the first trimester, in other Mediterranean countries, figures were similar or lower in Spain 95 *μ*g/L [21] and 125 *μ*g/L [22], and in Italy (115 in long-term supplementation versus 63 *μ*g/L in short-term supplementation [23]). However, UIE was higher in Switzerland (267 *μ*g/L) where an active program of supplementation exists [24, 25]. On the other hand, in Belgium, UIE was very low (36 *μ*g/L) in a selected population of women with excessive thyroid stimulation [10]. Based on UIE, in our study of selected women with normal thyroid function in early pregnancy, ID was present in 66% of cases, using a threshold of 150 *μ*g/L. This prevalence was similar to those many studies worldwide performed at the same gestational age, except for Brander in Switzerland [24].

UIE did not correlate with any parameters of maternal thyroid function at this stage of pregnancy in our selected healthy population. We had already reported this lack of correlation in our previous study performed during the third trimester [2]. This has also been reported by Fuse et al. [26] in a large population in an area of sufficient iodine intake, though women with excessive intake (UIE > 1 mg/L) had higher TSH [26]. In a population with moderate-to-severe iodine deficiency, it is usually reported a relative hypothyroxinemia, with or without elevation of TSH, particularly in women with predisposition to thyroid disease [8]. On the other hand, Orito et al. had shown in early pregnancy a positive correlation of UIE with TSH and a negative correlation with fT4 and fT3 in an area of excess iodine intake [27]. This suggests a U-shape curve with higher TSH observed in case of iodine deficiency and in areas of iodine excess. To some degree, this is true with thyroid volume as well. UIE in our patients correlated negatively with thyroid volume as measured by ultrasound, the smaller volume of thyroid being noted in women with adequate iodine intake. An inverse relationship between UIE and thyroid volume has also been well documented in schoolchildren in Europe, including France [28].

 In contrast with UIE, *β*hCG correlated strongly with thyroid tests. As expected, we found a negative correlation of *β*hCG and TSH, reflecting the classical TSH-like activity of *β*hCG [8]. *β*HCG correlated positively with all the tested parameters, including rT3, except for fT3. Thus, *β*hCG appears as the main driver of thyroid function at this stage of pregnancy, as also suggested by Haddow [29]. We report here a correlation of *β*HCG with rT3. Interestingly, Asakura et al. have reported a correlation of rT3 with the severity of hyperemesis gravidarum [30], a condition generally associated with high *β*hCG levels. This could suggest a shift in deiodinase activity in that condition. 

Of note, we report a strong correlation of *β*hCG with UIE. One physiological explanation could be that *β*hCG stimulates relaxin secretion by the corpus luteum, which plays an important physiologic role in the maternal renal adaptation to pregnancy [31]. We speculate that this could contribute to gestational increased UIE during the first trimester. 

Maternal smoking is recognized as a stress on the thyroid [32]. The higher thyroid volume and higher thyroglobulin levels we report here in smokers illustrate the deleterious impact of tobacco on the thyroid. We found no correlation between maternal smoking and UIE, though median UIE was slightly lower in smokers (data not shown). Importantly, there was no correlation as well between Tg and UIE. This shows that Tg is more likely a marker of maternal smoking than iodine deficiency at this stage of pregnancy in an area of mild iodine deficiency. However, later in pregnancy, maternal smoking has a significant impact on CB Tg only, but not on maternal Tg [33]. The strong negative correlation of smoking with rT3 has not been reported before and could suggest an effect of tobacco on deiodinase activity. In contrast, we found no correlation of maternal smoking with other thyroid parameters. Shields et al. had found correlations during the first trimester with TSH (lower), and fT3 (higher), and no correlation with fT4 [34]. There was no information on Tg nor thyroid volume, nor rT3. On the other hand, Pearce et al. found that fT4 was lower in women smoking during pregnancy. They did not find association of TSH with smoking or the use of iodine containing vitamins [35]. The impact of other toxics, such as known endocrine disruptors (e.g., PCBs) could also play a role in pregnancy [36, 37].

## 5. Conclusion

In pregnant women of our area selected for a strictly normal thyroid function, ID is present in two-third of women during the 1st trimester, though usually mild. The absence of correlation between UIE and thyroid tests at that stage of pregnancy contrasts with the impact of *β*HCG that appears as the main driver of maternal thyroid function in early pregnancy, possibly overriding ID. In addition, our study illustrates the impact of maternal smoking on thyroid volume, Tg, and rT3.

## Figures and Tables

**Table 1:** Ioduria and thyroid tests performed at 8 weeks of amenorrhea (5–12) in the 110 included women.

	Median	Mean ± SD	Min	Max
Ioduria *μ*g/L	116	139 ± 98	<15	399
fT4 pmol/L	14.35	14.57 ± 1.65	12.0	19.16
TSH mUI/L	1.18	1.24 ± 0.58	0.13	2.47
fT3 pmol/L	5.0	5.1 ± 0.5	3.8	6.7
TT4 nmol/L	110.7	111.1 ± 23	50.7	167.4
TBG *μ*g/mL	26.7	28.1 ± 7.2	12.8	50
Tg ng/mL	17.4	22.3 ± 17.2	1.3	100
*β*hCG UI/L	62724	65289 ± 47845	1025	220731
rT3 nmol/L	0.27	0.27 ± 0.07	0.13	0.51

**Table 2:** Main significant correlations.

Ioduria			
Negatively	Maternal age	*r* = −0.2	*P* = 0.05
	Thyroid volume	*r* = −0.22	*P* = 0.03
Positively	*β*HCG	*r* = 0.27	*P* < 0.01

TSH			
Negatively	fT4	*r* = −0.27	*P* < 0.005
	rT3	*r* = −0.24	*P* = 0.01
	*β*hCG	*r* = −0.36	*P* < 0.0001
Positively	Maternal weight	*r* = 0.19	*P* = 0.06

fT4			
Negatively	Maternal weight	*r* = −0.2	*P* = 0.05
Positively	TT4	*r* = 0.44	*P* < 0.0001
	rT3	*r* = 0.44	*P* < 0.0001
	*β* hCG	*r* = 0.18	*P* = 0.058

fT3			
Positively	Tg	*r* = 0.22	*P* = 0.02

rT3			
Negatively	Smoking		*P* = 0.001
Positively	*β*hCG	*r* = 0.4	*P* < 0.0001
	fT4	*r* = 0.44	*P* < 0.0001
	TT4	*r* = 0.5	*P* < 0.0001
	TBG	*r* = 0.33	*P* = 0.0005

Tg			
Positively	fT3	*r* = 0.22	*P* = 0.02
	Smoking		*P* = 0.0001

*β*hCG			
Negatively	TSH	*r* = −0.36	*P* < 0.0001
Positively	Ioduria	*r* = 0.27	*P* < 0.01
	TT4	*r* = 0.3	*P* < 0.005
	fT4	*r* = 0.18	*P* = 0.058
	rT3	*r* = 0.4	*P* < 0.0001
	TBG	*r* = 0.40	*P* < 0.0001

Thyroid volume			
Negatively	Ioduria	*r* = −0.22	*P* = 0.05
Positively	Smoking		*P* = 0.02

## Abbreviations

BMI: Body mass index
CV: Coefficient of variation
fT3: Free T3
fT4: Free T4
ID: Iodine deficiency
rT3: Reverse T3
RIA: Radio-immunoassay
TBG: Thyroxin Binding Globulin
Tg: Thyroglobulin
UIE: Urinary iodine excretion
WA: Weeks of amenorrhea.

## References

[1] Soldin OP (2002). Controversies in urinary iodine determinations. *Clinical Biochemistry*.

[2] Hiéronimus S, Bec-Roche M, Ferrari P, Chevalier N, Fénichel P, Brucker-Davis F (2009). Iodine status and thyroid function of 330pregnant women from Nice area assessed during the second part of pregnancy. *Annales d’Endocrinologie*.

[3] Delange F (2001). Iodine deficiency as a cause of brain damage. *Postgraduate Medical Journal*.

[4] Zimmermann MB (2007). The adverse effects of mild-to-moderate iodine deficiency during pregnancy and childhood: a review. *Thyroid*.

[5] Morreale De Escobar G, Obregon MJ, Del Rey FE (2000). Is neuropsychological development related to maternal hypothyroidism or to maternal hypothyroxinemia?. *The Journal of Clinical Endocrinology & Metabolism*.

[6] De Escobar GM, Obregón MJ, Del Rey FE (2007). Iodine deficiency and brain development in the first half of pregnancy. *Public Health Nutrition*.

[7] Pop VJ, Kuijpens JL, Van Baar AL (1999). Low maternal free thyroxine concentrations during early pregnancy are associated with impaired psychomotor development in infancy. *Clinical Endocrinology*.

[8] Glinoer D (1997). The regulation of thyroid function in pregnancy: pathways of endocrine adaptation from physiology to pathology. *Endocrine Reviews*.

[9] Mandel SJ, Spencer CA, Hollowell JG (2005). Are detection and treatment of thyroid insufficiency in pregnancy feasible?. *Thyroid*.

[10] Glinoer D, De Nayer P, Delange F (1995). A randomized trial for the treatment of mild iodine deficiency during pregnancy: maternal and neonatal effects. *The Journal of Clinical Endocrinology & Metabolism*.

[11] Velasco I, Carreira M, Santiago P (2009). Effect of iodine prophylaxis during pregnancy on neurocognitive development of children during the first two years of life. *The Journal of Clinical Endocrinology & Metabolism*.

[12] Qian M, Wang D, Watkins WE (2005). The effects of iodine on intelligence in children: a meta-analysis of studies conducted in China. *Asia Pacific Journal of Clinical Nutrition*.

[13] Pearce EN (2009). What do we know about iodine supplementation in pregnancy?. *The Journal of Clinical Endocrinology & Metabolism*.

[14] Abalovich M, Amino N, Barbour LA (2007). Management of thyroid dysfunction during pregnancy and post partum : an Endocrine Society clinical practice guideline. *The Journal of Clinical Endocrinology & Metabolism*.

[15] Kang HW, No JH, Chung JH (2004). Prevalence, clinical and ultrasonographic characteristics of thyroid incidentalomas. *Thyroid*.

[16] Andersson M, De Benoist B, Delange F, Zupan J (2007). Prevention and control of iodine deficiency in pregnant and lactating women and in children less than 2-years-old: conclusions and recommendations of the Technical Consultation. *Public Health Nutrition*.

[17] ICCIDD Newsletter (2007). Iodine requirements in pregnancy and infancy. *International Council For the Control of Iodine Deficiency Disorders*.

[18] Stilwell G, Reynolds PJ, Parameswaran V, Blizzard L, Greenaway TM, Burgess JR (2008). The influence of gestational stage on urinary iodine excretion in pregnancy. *The Journal of Clinical Endocrinology & Metabolism*.

[19] Laurberg P, Andersen S, Bjarnadóttir RI (2007). Evaluating iodine deficiency in pregnant women and young infants—complex physiology with a risk of misinterpretation. *Public Health Nutrition*.

[20] Caron P, Hoff M, Bazzi S (1997). Urinary iodine excretion during normal pregnancy in healthy women living in the southwest of France: correlation with maternal thyroid parameters. *Thyroid*.

[21] Alvarez-Pedrerol M, Guxens M, Mendez M (2009). Iodine levels and thyroid hormones in healthy pregnant women and birth weight of their offspring. *European Journal of Endocrinology*.

[22] Sánchez-Vega J, del Rey FE, Fariñas-Seijas H, de Escobar GM (2008). Inadequate iodine nutrition of pregnant women from Extremadura (Spain). *European Journal of Endocrinology*.

[23] Moleti M, Presti VPL, Campolo MC (2008). Iodine prophylaxis using iodized salt and risk of maternal thyroid failure in conditions of mild iodine deficiency. *The Journal of Clinical Endocrinology & Metabolism*.

[24] Brander L, Als C, Buess H (2003). Urinary iodine concentration during pregnancy in an area of unstable dietary iodine intake in Switzerland. *Journal of Endocrinological Investigation*.

[25] Zimmermann MB, Aeberli I, Torresani T, Bürgi H (2005). Increasing the iodine concentration in the Swiss iodized salt program markedly improved iodine status in pregnant women and children: a 5-y prospective national study. *The American Journal of Clinical Nutrition*.

[26] Fuse Y, Ohashi T, Yamaguchi S, Yamaguchi M, Shishiba Y, Irie M (2011). Iodine status of pregnant and post partum Japanese women: effect of iodine intake on maternal and neonatal thyroid function in an iodine sufficient area. *The Journal of Clinical Endocrinology & Metabolism*.

[27] Orito Y, Oku H, Kubota S (2009). Thyroid function in early pregnancy in Japanese healthy women: relation to urinary iodine excretion, emesis, and fetal and child development. *The Journal of Clinical Endocrinology & Metabolism*.

[28] Delange F, Benker G, Caron P (1997). Thyroid volume and urinary iodine in European schoolchildren: standardization of values for assessment of iodine deficiency. *European Journal of Endocrinology*.

[29] Haddow JE, McClain MR, Lambert-Messerlian G (2008). Variability in thyroid-stimulating hormone suppression by human chronic gonadotropin during early pregnancy. *The Journal of Clinical Endocrinology & Metabolism*.

[30] Asakura H, Watanabe S, Sekiguchi A, Power GG, Araki T (2000). Severity of hyperemesis gravidarum correlates with serum levels of reverse T3. *Archives of Gynecology and Obstetrics*.

[31] Smith MC, Murdoch AP, Danielson LA, Conrad KP, Davison JM (2006). Relaxin has a role in establishing a renal response in pregnancy. *Fertility and Sterility*.

[32] Kapoor D, Jones TH (2005). Smoking and hormones in health and endocrine disorders. *European Journal of Endocrinology*.

[33] Hieronimus S, Ferrari P, Gal J Relative impact of iodine supplementation and maternal smoking on cord blood thyroglobulin in pregnant women with normal thyroid function.

[34] Shields B, Hill A, Bilous M (2009). Cigarette smoking during pregnancy is associated with alterations in maternal and fetal thyroid function. *The Journal of Clinical Endocrinology & Metabolism*.

[35] Pearce EN, Oken E, Gillman MW (2008). Association of first-trimester thyroid function test values with thyroperoxidase antibody status, smoking, and multivitamin use. *Endocrine Practice*.

[36] Zoeller RT (2007). Environmental chemicals impacting the thyroid: targets and consequences. *Thyroid*.

[37] Brucker-Davis F, Ferrari P, Boda-Buccino M (2011). Cord blood thyroid tests in boys born with and without cryptorchidism. Correlations with birth parameters and in utero xenobiotics exposure. *Thyroid*.

